# Changes in the gut microbiota composition during pregnancy in patients with gestational diabetes mellitus (GDM)

**DOI:** 10.1038/s41598-018-30735-9

**Published:** 2018-08-15

**Authors:** Ilario Ferrocino, Valentina Ponzo, Roberto Gambino, Adriana Zarovska, Filomena Leone, Clara Monzeglio, Ilaria Goitre, Rosalba Rosato, Angelo Romano, Giorgio Grassi, Fabio Broglio, Maurizio Cassader, Luca Cocolin, Simona Bo

**Affiliations:** 1 Department of Agricultural, Forest and Food Sciences, University of Turin, Turin, Italy; 2 Department of Medical Sciences, University of Turin, Turin, Italy; 3 Clinical Nutrition Unit, S. Anna Hospital, Città della Salute e della Scienza, Turin, Italy; 4 Gynecology and Obstetrics Unit, S. Anna Hospital, Città della Salute e della Scienza, Turin, Italy; 5 Department of Psychology, University of Turin, Turin, Italy; 6 SC Controllo Alimenti e Igiene delle Produzioni, Istituto Zooprofilattico Sperimentale PVL, Turin, Italy

## Abstract

Gestational diabetes mellitus (GDM), a common pregnancy complication, is associated with an increased risk of maternal/perinatal outcomes. We performed a prospective observational explorative study in 41 GDM patients to evaluate their microbiota changes during pregnancy and the associations between the gut microbiota and variations in nutrient intakes, anthropometric and laboratory variables. GDM patients routinely received nutritional recommendations according to guidelines. The fecal microbiota (by 16S amplicon-based sequencing), was assessed at enrolment (24–28 weeks) and at 38 weeks of gestational age. At the study end, the microbiota α-diversity significantly increased (*P* < 0.001), with increase of Firmicutes and reduction of Bacteroidetes and Actinobacteria. Patients who were adherent to the dietary recommendations showed a better metabolic and inflammatory pattern at the study-end and a significant decrease in *Bacteroides*. In multiple regression models, *Faecalibacterium* was significantly associated with fasting glucose; *Collinsella* (directly) and *Blautia* (inversely) with insulin, and with Homeostasis-Model Assessment Insulin-Resistance, while *Sutterella* with C-reactive protein levels. Consistent with this latter association, the predicted metagenomes showed a correlation between those taxa and inferred KEGG genes associated with lipopolysaccharide biosynthesis. A higher bacterial richness and strong correlations between pro-inflammatory taxa and metabolic/inflammatory variables were detected in GDM patients across pregnancy. Collectively these findings suggest that the development of strategies to modulate the gut microbiota might be a potentially useful tool to impact on maternal metabolic health.

## Introduction

Gestational diabetes mellitus (GDM), one of the most common pregnancy complications, is associated with a moderately increased risk of maternal and perinatal outcomes^1,2^. Lifestyle interventions were reported to provide benefits to the health of GDM women and their babies^1^. It has been hypothesized that at least some of these beneficial effects might be due to the modulation of the maternal microbiota during pregnancy^3–7^. Indeed, variations in nutrient and energy intake were associated to specific bacterial abundance^8–10^. During the course of normal pregnancy, gut microbiota has been reported to remain relatively stable^11^ or to change dramatically, with an increase in Proteobacteria and Actinobacteria, a decline in butyrate-producing bacteria, a reduction in bacterial richness and within-subject (α) diversity, and higher between-subject (β) diversity at the end of pregnancy^3^. These modifications were supposed to favor the metabolic changes which support the healthy fetal growth, such as reduced insulin sensitivity and increased nutrient absorption^3,7^. Only few studies have evaluated the microbiota of GDM patients, showing contrasting results: either no differences^3^, decreased placental abundance of the Pseudomonadales order and *Acinetobacter* genus^11^, or increased placental Proteobacteria and reduced placental Bacteroidetes and Firmicutes^12^ have been reported in comparison with normoglycemic mothers. In addition, dysbiosis among GDM patients was reported to be associated with a few genus belonging to Firmicutes, Bacteroides and Actinobacteria phyla of the gut microbiota and the observed main differences in comparison to healthy women are relative to the gene contents of the gut microbes^13^. Two studies performed in women with previous GDM showed a relatively higher stool abundance of the *Prevotellaceae* family and a reduced abundance of the Firmicutes phylum^14^, or no differences in the gut microbiota composition^15^ in comparison to normoglycemic post-partum controls. Furthermore, increased gut relative abundance of the *Ruminococcaceae* family was associated with higher odds of developing GDM^16^. Finally, the cross-sectional design did not allow to draw conclusions about the causal relationships of the associations found.

In consideration of these highly divergent results, mostly derived from cross-sectional studies, we aimed to perform a prospective observational study evaluating the dynamic changes of the microbiota occurring during pregnancies of GDM women. All patients, after the diagnosis of GDM, underwent an educational dietary intervention, according to guidelines^17^, but the compliance with the provided recommendations is variable between women.

Therefore, the aims of our study were evaluating: i) whether the within-patient gut microbiota composition varied from the second to the third trimester of pregnancy; ii) whether patients with greater adherence to dietary recommendations presented a different microbial pattern than the less adherent ones; iii) whether changes in microbiota composition were associated with variations in nutrient intakes, anthropometric and laboratory variables; iv) whether specific microbiota oligotypes were implicates in these associations.

## Results

### Characteristics of the participants

Nine women did not return stool samples and were lost at follow-up. Data of 41 patients were therefore analyzed. The clinical characteristics of the participants did not differ from those of the 9 women who dropped out (data not shown).

Seven women (17.1%) gave birth before the 38^th^ week. These patients provided the fecal and blood samples and the food questionnaire about a week before all the others (37^th^ week); they did not differ with regard to nutritional, anthropometric, or metabolic characteristics when compared to the others.

Most participants were overweight women, with excessive fat intake and lower than recommended fiber consumption. From enrolment (24–28 weeks of gestational age) to the study end (38 weeks), weight and Body Mass Index (BMI) increased, and metabolic and inflammatory patterns of participants worsened, as usually occurs during the third trimester of pregnancy (Table 1).

**Table 1:** Characteristics of the participants at enrolment and at the study end.

	At enrolment	Study end	P*
Number	41	41	
Age	37.1 ± 4.2		
Pre-pregnancy weight (kg)	69.3 ± 14.6		
Pre-pregnancy BMI (kg/m^2^)	25.8 ± 5.9		
Nulliparous (%)	58.5		
***Education (%)***			
Primary school	17.1		
Secondary school	41.5		
University degree	41.5		
***Anthropometric and Blood measurements***			
METS (h/week)	27.0 (36.4)	27.0 (26.5)	0.74**
Weight (kg)	75.8 ± 12.9	79.0 ± 13.3	**<0.001**
BMI (kg/m^2^)	28.2 ± 5.3	29.4 ± 5.4	**<0.001**
Systolic BP (mmHg)	110.8 ± 11.7	116.1 ± 11.6	0.02
Diastolic BP (mmHg)	72.9 ± 7.5	75.8 ± 9.1	0.07
Fasting glucose (mg/dL)	97.9 ± 19.2	96.6 ± 19.1	0.57
HbA1c (%)	4.6 ± 0.8	4.9 ± 0.8	0.06
Fasting insulin (µU/mL)	10.1 (8.4)	11.6 (10.0)	0.02**
HOMA-IR (mmol/L*µU/mL)	2.3 (1.9)	2.8 (2.7)	0.15**
Total cholesterol (mg/dL)	234.1 ± 32.4	257.0 ± 48.6	**<0.001**
HDL-cholesterol (mg/dL)	65.8 ± 13.4	67.0 ± 15.6	0.54
Triglycerides (mg/dL)	173.3 ± 53.0	259.2 ± 70.5	**<0.001**
CRP (mg/L)	4.1 (4.2)	4.5 (7.5)	0.007**
***Dietary intakes***			
Energy (kcal)	1605.8 ± 254.4	1766.1 ± 306.7	0.009
Carbohydrates (% total kcal)	44.4 ± 6.6	43.1 ± 6.4	0.27
Sugars (% total kcal)	8.8 ± 4.7	6.2 ± 4.5	0.008
Sugars (g/day)	35.3 ± 20.1	27.9 ± 21.3	0.08
Oligosaccharides (g/day)	36.7 ± 19.7	54.2 ± 23.2	<0.001
Starch (g/day)	107.3 ± 28.9	109.7 ± 38.7	0.73
Fiber (g/day)	14.5 ± 4.2	15.1 ± 5.3	0.48
Proteins (% total kcal)	15.6 ± 2.3	16.6 ± 5.3	0.22
Total fats (% total kcal)	42.2 ± 5.2	42.3 ± 6.3	0.89
SFA (% total kcal)	11.3 ± 2.2	11.1 ± 2.7	0.65
PUFA (%kcal)	4.9 ± 1.7	4.4 ± 1.1	0.09
***Pregnancy outcomes***			
Insulin treatment (%)		9.8	
Cesarean section (%)		24.4	
Gestational age at delivery (weeks)		39.2 ± 1.2	
LGA newborns (%)		9.8	
Male newborns (%)		53.7	

BMI = body mass index, METS = metabolic equivalent of activity, BP = blood pressure, HbA1c = glycated hemoglobin, HOMA-IR = Homeostasis Model Assessment-Insulin Resistance, HDL = high density lipoprotein, LDL = low-density lipoprotein, CRP = C-reactive protein, SFA = saturated fatty acids, PUFA = polyunsaturated fatty acids, LGA = large-for-gestational age. Values are expressed as mean ± standard deviation or median (interquartile range) ^*^Paired-sample *t*-test, ^**^Wilcoxon matched pairs test.

### Adherence to the dietary recommendations

After the dietary counselling, 34.1% (14/41) of the participants declared to be adherent to the given dietary recommendations. Characteristics at enrolment did not significantly differ between adherents and non-adherents, even if adherents showed increased values of weight and BMI (Table 2). Adherent women showed reduced intakes of sugars, and increased consumption of fiber, oligosaccharides, polyunsaturated fatty acids (PUFA) than non-adherents (Table 2). All participants had abolished alcohol consumption. Adherents had a better metabolic and inflammatory pattern, with a significantly greater reduction in fasting glucose and Homeostasis Model Assessment-Insulin Resistance (HOMA-IR) levels at the end of the study.

**Table 2:** Characteristics of the participants by adherence to the lifestyle recommendations and median changes from enrolment (deltas).

	Baseline			Study End			Delta		
	Adherent	Not adherent	*P*	Adherent	Not adherent	*P*	Adherent	Not adherent	P*
Number	14	27		14	27		14	27	
Age	35.5 ± 3.8	38.0 ± 4.3	0.08						
Pre-pregnancy weight (kg)	73.1 ± 18.0	67.4 ± 12.5	0.24						
Pre-pregnancy BMI (kg/m^2^)	28.0 ± 8.0	24.7 ± 4.3	0.09						
Nulliparous (%)	64.3	55.6	0.27						
***Education*** (***%***)									
Secondary school	42.9	40.7							
University degree	42.9	40.7	0.94						
METS (h/week)	32.3 (37.0)	24.5 (38.0)	0.39*	27.9 (31.5)	23.3 (32.3)	0.08*	0.0	0.0	0.17
Weight (kg)	78.6 ± 16.7	74.3 ± 10.4	0.31	80.9 ± 17.0	77.9 ± 11.2	0.50	+ 2.0	+ 3.0	0.10
BMI (kg/m^2^)	30.1 ± 7.4	27.2 ± 3.6	0.10	30.9 ± 7.4	28.6 ± 3.8	0.18	+ 0.7	+ 1.2	0.10
Systolic BP (mmHg)	111.7 ± 12.8	110.3 ± 11.4	0.73	117.1 ± 12.2	115.6 ± 11.5	0.70	+ 7.0	+ 4.0	0.61
Diastolic BP (mmHg)	71.8 ± 9.0	73.5 ± 6.6	0.49	77.9 ± 10.2	74.7 ± 8.5	0.29	+ 7.0	0.0	0.21
Fasting glucose (mg/dL)	99.8 ± 29.3	96.9 ± 11.4	0.65	88.9 ± 25.3	100.6 ± 13.8	0.06	−6.0	+ 1.0	**<0.001**
**Post**-**prandial glucose** (**mg**/**dL**)				106.3 ± 8.3	118.1 ± 12.9	0.004			
HbA1c (%)	4.8 ± 0.9	4.6 ± 0.8	0.42	4.8 ± 0.8	5.0 ± 0.8	0.40	+ 0.1	+ 0.5	0.19
Fasting insulin (µU/mL)	11.3 (11.3)	9.0 (6.1)	0.83*	11.4 (10.8)	11.6 (12.4)	0.66*	−0.20	+ 2.0	0.003
HOMA-IR (mmol/L*µU/mL)	2.7 (2.8)	2.1 (1.3)	0.19*	2.4 (3.0)	3.1 (2.5)	0.38*	−0.45	+ 0.47	**<0.001**
Total cholesterol (mg/dL)	227.1 ± 33.1	237.8 ± 32.1	0.32	246.3 ± 41.5	262.6 ± 51.7	0.32	+ 20.0	+ 27.0	0.82
HDL-cholesterol (mg/dL)	68.0 ± 11.5	64.6 ± 14.4	0.45	68.9 ± 12.4	66.0 ± 17.1	0.58	+ 1.0	+ 1.0	0.73
Triglycerides (mg/dL)	159.1 ± 57.4	180.6 ± 50.1	0.22	246.5 ± 81.7	265.9 ± 64.5	0.41	+ 76.5	+ 91.0	0.65
CRP (mg/L)	3.2 (5.2)	4.3 (4.4)	0.76*	3.2 (3.1)	8.4 (8.3)	0.008*	−0.02	+ 2.5	0.003
***Dietary intakes***									
Energy (kcal)	1659.2 ± 309.6	1578.1 ± 222.0	0.34	1828.6 ± 200.7	1733.6 ± 348.5	0.35	+ 161.5	+ 88.0	0.44
Carbohydrates (% total kcal)	44.2 ± 5.0	44.5 ± 7.3	0.90	43.0 ± 5.1	43.2 ± 7.1	0.92	−2.0	−2.0	0.74
Sugars (% total kcal)	9.7 ± 3.7	8.3 ± 5.1	0.36	3.9 ± 2.2	7.5 ± 5.0	0.015	−6.9	−1.5	0.005
Sugars (g/day)	40.7 ± 18.4	32.5 ± 20.8	0.22	17.5 ± 9.7	33.4 ± 23.7	0.02	−20.2	−7.6	0.004
Oligosaccharides (g/day)	39.7 ± 19.8	35.2 ± 19.9	0.50	66.7 ± 22.6	47.8 ± 21.1	0.01	+ 14.7	+ 13.8	0.41
Starch (g/day)	104.3 ± 24.3	108.8 ± 31.3	0.64	112.9 ± 44.9	108.1 ± 35.9	0.71	+ 23.1	−13.8	0.33
Fiber (g/day)	15.2 ± 5.4	14.2 ± 3.5	0.43	20.5 ± 2.1	12.4 ± 4.2	**<0.001**	+ 5.8	−0.94	**<0.001**
Proteins (% total kcal)	15.9 ± 1.6	15.4 ± 2.6	0.48	19.4 ± 6.4	15.2 ± 4.1	0.016	+ 1.9	−0.1	0.26
Total fats (% total kcal)	41.7 ± 4.2	42.4 ± 5.7	0.67	39.7 ± 5.4	43.7 ± 6.4	0.06	−1.4	+ 1.1	0.39
SFA (% total kcal)	11.6 ± 2.4	11.2 ± 2.2	0.60	9.7 ± 1.6	11.8 ± 2.9	0.017	−3.0	+ 0.6	0.03
PUFA (%kcal)	5.2 ± 2.6	4.7 ± 1.1	0.32	5.0 ± 1.1	4.0 ± 0.9	0.003	+ 0.1	−0.5	0.07
***Pregnancy outcomes***									
Insulin treatment (%)				7.1	11.1	0.68**			
Cesarean section (%)				21.4	25.9	0.75**			
Gestational age at delivery (weeks)				39.2 ± 1.3	39.2 ± 1.2	0.91			
LGA newborns (%)				7.1	11.1	0.68**			
Male newborns (%)				50.0	55.6	0.74**			

BMI = body mass index, METS = metabolic equivalent of activity, BP = blood pressure, HbA1c = glycated hemoglobin, HOMA-IR = Homeostasis Model Assessment-Insulin Resistance, HDL = high density lipoprotein, LDL = low-density lipoprotein, CRP = C-reactive protein, SFA = saturated fatty acids, PUFA = polyunsaturated fatty acids, LGA = large-for-gestational age.

Values are expressed as mean ± standard deviation or median (interquartile range); deltas = median values of the following difference: (end-of the study values minus baseline values).

*P*-values were calculated by *t*-student test or chi-square test; ^*^P-values by Mann-Whitney test; ^**^P-values by Chi-square test.

### Microbiota composition at enrolment and at the study end

**Figure 1:**
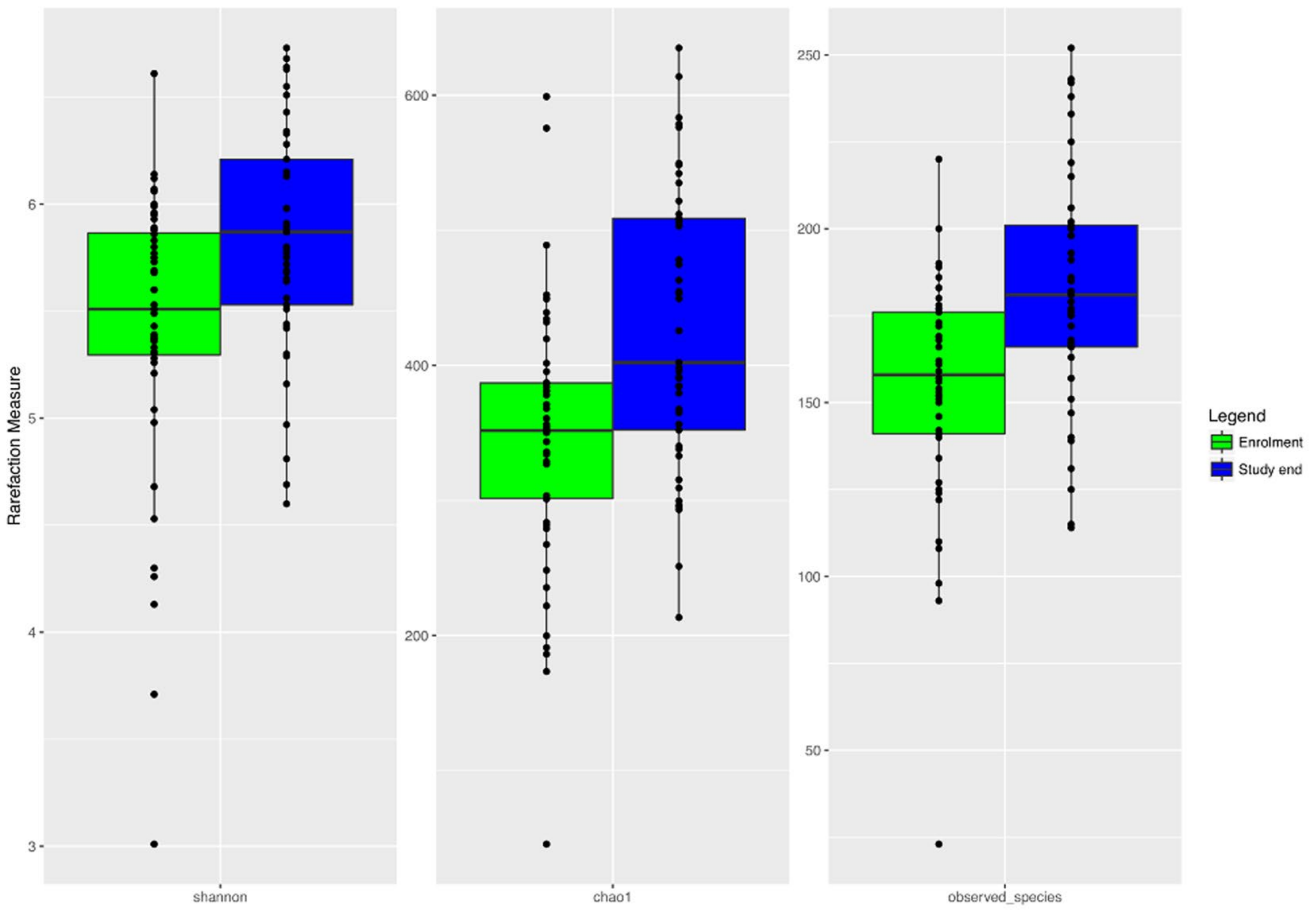
Boxplots to describe α-diversity measures of fecal microbiota of GDM patients at enrolment (green bars) and study end (blue bars). Individual points and brackets represent the richness estimate and the theoretical standard error range, respectively.

**Figure 2:**
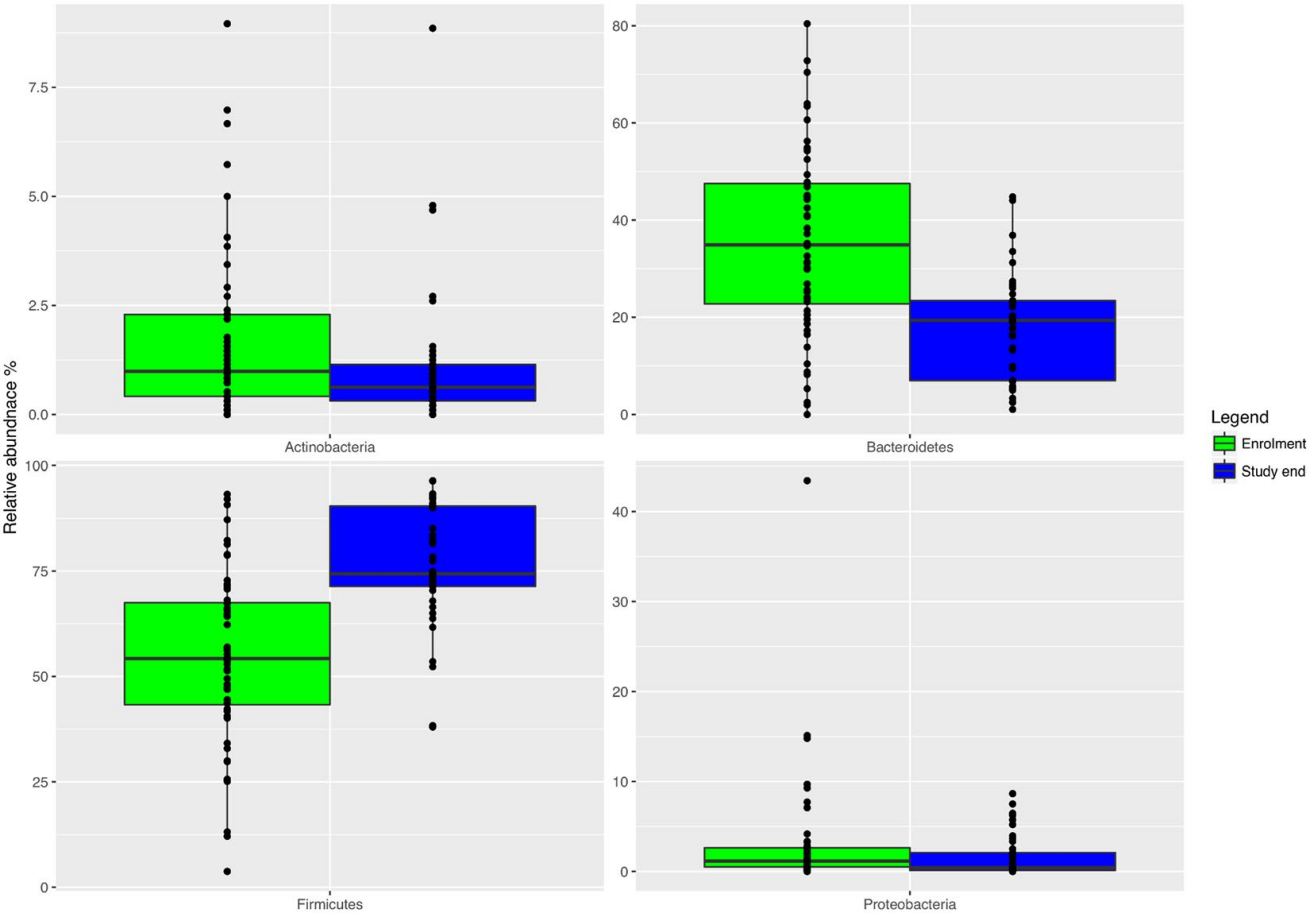
Boxplots showing the relative abundance of Actinobacteria, Bacteroidetes, Proteobacteria and Firmicutes phyla in fecal samples of GDM patients at enrolment (green bars) and study end (blue bars). Boxes represent the interquartile range (IQR) between the first and third quartiles, and the line inside represents the median (2nd quartile). Whiskers denote the lowest and the highest values within 1.56 IQR from the first and third quartiles, respectively. Circles represent outliers beyond the whiskers.

**Figure 3:**
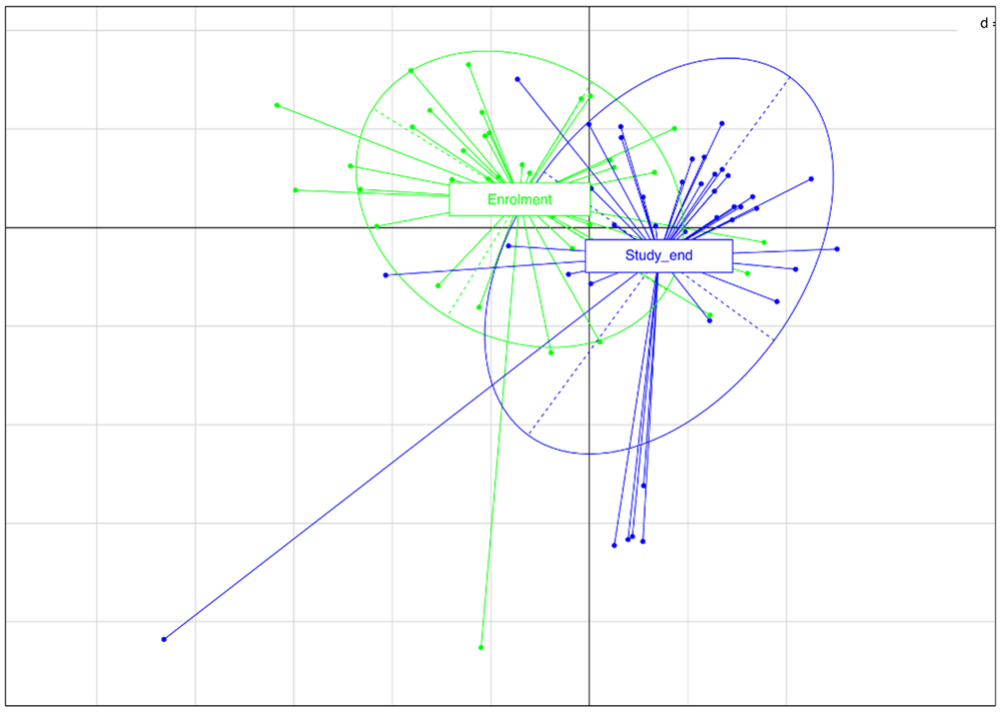
Principal Component Analysis (PCA) based on OTUs relative abundance of GDM patients at enrolment (green) and study end (blue). The first component (horizontal) accounts for the 22.9% of the variance and the second component (vertical) accounts for the 23.5%.

**Figure 4:**
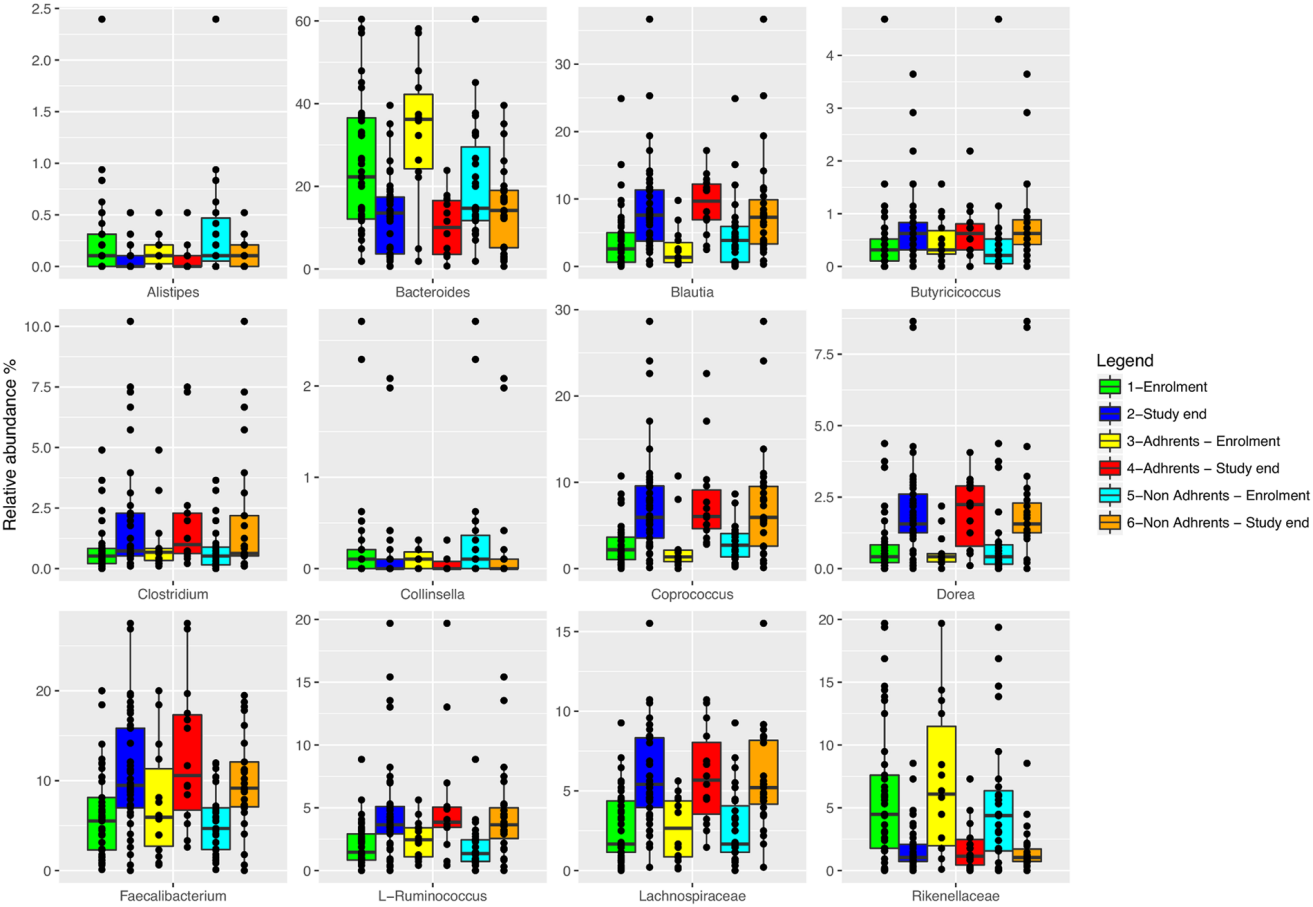
Boxplots showing the relative abundance at genus or family level of the OTUs differentially abundant based on Wilcoxon matched pairs test (*P* ≤ 0.002) in fecal samples between: GDM patients at enrolment (green bars) and at the study end (blue bars); adherents to the dietary recommendations at enrolment (yellow bars) and at the study end (red bars); non-adherents to the dietary recommendations at enrolment (cyan bars) and at the study end (orange bars).

The microbiota α-diversity values were significantly different between subjects at enrolment when compared to subjects at the end of the study (*P* < 0.001). In details, species richness, number of different species and the Shannon index were significantly higher at the end of the study (*P* < 0.001) (Fig. 1). The analysis of microbial taxa abundance at phylum level showed an increase of Firmicutes at the study end, and a reduction of Actinobacteria and Bacteroidetes (Fig. 2). Going more deeply in the microbial composition, the level of diversity of the subjects based on the structure of their microbiota was clearly different across time (Supplementary Fig. S1). Moreover, Principal Component Analysis (PCA) based on microbiota composition (Fig. 3) revealed a significant relationship between genus-level microbiota composition and sampling time confirmed by ADONIS and ANOSIM statistical test (*P* < 0.001). Boxplot at genus level (Fig. 4) showed a significant reduction in the abundance of *Bacteroides*, *Collinsella* and *Rikenellaceae*, and a significant increase of *Blautia*, *Butyricicoccus*, *Clostridium*, *Coprococcus*, *Dorea*, *Faecalibacterium*, L−*Ruminococcus* (*Ruminococcus* genus assigned to *Lachnospiraceae* family), and *Lachnospiraceae* at the study end when compared to enrolment (Fig. 4).

### Microbiota signature between dietary adherences

The microbiota α- and β-diversity values were not significantly different between adherent and non-adherent subjects (data not shown). Similarly, there was no significant separation of the microbiota composition. We performed a number of analyses investigating the shift in microbiota as a function of the adherence to diet. Taking into account the shift in the microbiota between enrolment and study end in adherents and non-adherents (Fig. 4) a common microbiota signature was observed. *Blautia, Coprococcus, Dorea* and *Lachnospiraceae* significantly increased in both groups during the progression of pregnancy while *Rikenellaceae* decreased. We observed that the delta (study-end minus baseline) values of those OTUs was significantly higher in adherent patients. Between the two groups, we detected a specific microbiota shift at the study end: an impressive decrease in *Bacteroides* in adherents, and higher abundance of *Faecalibacterium* and L-*Ruminococcus* together with minor OTUs in non-adherents (Fig. 4).

### Associations between microbiota and nutrient intakes and metabolic variables

Several different associations between nutrients/metabolic variables and microbiota could be detected both at enrolment (Supplementary Fig. S2A) and at the study end (Supplementary Fig. S2A).

At enrolment, *Alistipes* was found positively related with fat intakes (β = 0.10; 95% CI 0.06 0.14; *P* < 0.001) in a regression model, after adjusting for age and weight values. Furthermore, glycated hemoglobin (HbA1c) levels correlated with both *Bacteroidales* (β = 1.43; 95% CI 0.67 2.19; *P* < 0.001) and *Prevotella* (β = 0.11; 95% CI 0.06 0.16; *P* < 0.001).

At the end of the study, many associations among specific microbiota relative abundance and nutrient intakes, metabolic and inflammatory variables and their changes across pregnancy were detected in multiple regression analyses, after adjusting for age, weight change, and adherence to the given recommendations (Table 3). Among the relationships, we underline the direct associations between *Roseburia* and fiber intake (β = 0.09; 95% CI 0.02 0.16; *P* = 0.01), and that between *L-Ruminococcus* and oligosaccharides (β = 0.02; 95% CI 0.01 0.03; *P* = 0.006), that however did not reach the established statistical cut-offs. Furthermore, *Faecalibacterium* resulted inversely correlated with fasting glucose; *Collinsella* and *Blautia* were respectively directly and inversely associated with insulin and HOMA-IR values; *Blautia* was inversely correlated with HbA1c levels while *Sutterella* directly with CRP values (Table 3). Results did not change significantly, after adjusting for pre-pregnancy BMI, educational level and exercise (data not shown).

**Table 3:** Statistically significant associations between microbiota composition at the study end and dietary and metabolic variable by Spearman’s correlations (left) and multiple regression analyses (right).

	Rho	Beta	95% CI	*P*
Dietary intakes*				
**Proteins (% total kcal)**				
*Faecalibacterium*	0.32	0.08	0.04 0.12	**<0.001**
Metabolic variables**				
**Diastolic BP (mmHg)**				
*Oscillospira*	−0.44	−2.01	−3.11 −0.91	**<0.001**
*Rikenecellaceae*	−0.51	−2.74	−3.97 −1.51	**<0.001**
**Delta fasting glucose (mg/dL)**				
*Faecalibacterium*	−0.54	−1.28	−1.71 −0.85	**<0.001**
**Delta glycated hemoglobin (%)**				
*Blautia*	−0.51	−0.06	−0.10 −0.03	**0.001**
**Delta fasting insulin (µU/mL)**				
*Blautia*	−0.35	−0.42	−0.67 −0.17	**0.001**
*Butyricimonas*	0.41	36.1	14.7 57.5	**0.002**
*Collinsella*	0.45	8.69	6.00 11.4	**<0.001**
*Coprobacillus*	0.39	6.52	3.29 9.75	**<0.001**
Delta HOMA-IR (mmol/L*µU/mL)				
*Blautia*	−0.36	−0.11	−0.17 −0.05	**0.002**
*Butyricimonas*	0.51	11.2	6.50 15.9	**<0.001**
*Collinsella*	0.45	2.37	1.80 2.94	**<0.001**
*Erysipelotrichia*	0.37	1.87	1.09 2.65	**<0.001**
**Delta CRP (mg/L)**				
*Sutterella*	0.62	7.57	5.02 10.1	**<0.001**

BP = blood pressure.

^*^Multiple regression model evaluating the association between log-transformed bacteria relative amount (dependent variable) and the specific nutrient (independent variable), after adjusting for age, weight change

^**^Multiple regression model evaluating the association between BP and laboratory variables (dependent variables) and bacteria (independent variables) after adjusting for age, weight change, and adherence to the given recommendations. Each row is a model.

### Gut microbiota signature at sub-genus level

In order to explore the possible effects at sub-genus level, we carried out oligotyping on sequences of *Blautia* and *Roseburia* since these were the only genera changing over time that showed a Shannon entropy index sufficient to identify all nucleotide positions that would resolve the oligotypes. Specific *Blautia* oligotypes (identified as *Blautia wexlerae* by BLASTn match): B1, B2, B4, B9, B11, B18, B27, B32, B36 B42, B49, B51 and B53, identified as *Blautia luti* were more abundant at the study end, while B41 and B59 decreased with the progress of pregnancy (Supplementary Fig. S3). When plotting the correlation between those oligotypes and dietary intake and blood variables (Supplementary Fig. S4), different correlations were found. In a multiple regression model, B42 was associated directly with total cholesterol (β = 16.6; 95% CI 7.10 26.1; *P* = 0.002), and B42 inversely with diastolic blood pressure (β = −3.06; 95% CI −4.75 −1.37; *P* = 0.001).

*Roseburia* oligotypes R10, R24, R50, and R70 increased at the study end when compared to enrolment (data not shown). Among the different correlations found by Spearman’s nonparametric correlations, none resulted significantly different in the regression model.

### Shift in predicted metagenomes

The pathway enrichment analysis of the predicted metagenomes showed an enrichment of KEGG orthologues, at study end when compared with baseline, of glycolysis/gluconeogenesis (ko00010), fructose and mannose metabolism (ko00051), galactose metabolism (ko00052), starch and sucrose metabolism (ko005009), biosynthesis of amino acids (ko01230), and a reduction of fatty acid metabolism (ko01212), biotin metabolism (ko00780) and folate biosynthesis (ko00790). When plotting the correlations between OTUs and inferred metabolic pathways, we observed a positive correlation between Lipopolysaccaride (LPS) biosynthesis (ko00540) with *Sutterella*, *Bacteroides* and *Phascolartobacterium* (Supplementary Fig. S5).

## Discussion

Our results showed a shift in the microbiota composition from the second to the third trimester of pregnancy, with higher α-diversity, Firmicutes increment, and Bacteroidetes and Actinobacteria reduction. Furthermore, associations between specific bacterial abundance and dietary and laboratory variables were detected.

The reduced insulin sensitivity of late pregnancy is considered beneficial to support fetal growth and increased nutrient absorption, even if it is associated with metabolic impairment and inflammation^3^. Women who developed GDM have greater reduction in insulin sensitivity and their insulin secretion is not sufficient to maintain euglycemia, leading to glucose intolerance^17^. This is counterintuitive owing to the progressive weight gain and increase in circulating levels of insulin, lipids, and inflammatory markers in the patients and the well-known association of low bacterial richness and adiposity, insulin resistance, dyslipidemia, and inflammatory phenotypes^18^. Going more deeply in the microbiota composition of our patients, we observed that the higher bacterial richness was related to Firmicutes. In a Finnish study, it has been reported that glycated hemoglobin values were positively associated with microbiota richness^19^ and mice transplanted with feces from obese and lean individuals showed a positive correlation of OTUs richness with both fasting insulin and HOMA-IR level^20^. The increase in Firmicutes abundance (and the reduction in *Bacteroidetes*) can be justified by the patients’ gestational weight gain, already overweight before pregnancy, not dissimilarly to what happens in obese patients^21,22^. Accordingly, the inferred metagenomic showed an increase in pathways involved in carbohydrate metabolism (in details, glycolysis/gluconeogenesis, fructose and mannose metabolism, galactose metabolism, and starch and sucrose metabolism) with the release of simply sugars due to the higher abundance of Firmicutes that harvested more energy from the diet^23^. It can be hypothesized that those enriched function could be related with the progressive weight gain and could be a feature in hyperglycemic phenomena.

Literature data are controversial: in normoglycemic pregnancy, weight gain was reported to be associated with *Escherichia coli*^24^ or *Bacteroides* abundance^25^ and both an increment in Proteobacteria and Actinobacteria and a decline in butyrate-producing bacteria (such as *Faecalibacterium*) were found^3^.

A higher Bacteroides-to-Firmicutes ratio has been found to correlate with elevated plasma glucose levels^26^. In our patients, however, the most relevant change was the weight gain at the end of pregnancy (Table 1). We detected a reduction in *Bacteroidetes* across pregnancy, but we found significant direct associations between *Bacteroidales* and *Prevotella* and HbA1c levels at enrolment.

Indeed, studies are difficult to compare due to the different ethnicity and food habits of the analyzed cohorts^27^ leading to inter-individual variations in the gut microbiota composition and the various methods used to analyze the microbiota, both causing results sometimes contradictory.

Further aspects of previous studies make the comparison with our results difficult, such as the fact that both normoglycemic and GDM women were combined together^3,7^, participants taking probiotics or antibiotics were not excluded^3^, dietary intakes did not change during pregnancy^3^, early pregnancy only was evaluated^3,7,28^.

Overall, our patients consumed a low-fiber and high-fat diet, an unhealthy dietary pattern which has been associated with GDM^1^. Most of them (about 2/3) did not change substantially their dietary habits after having received nutritional recommendations and showed a worse metabolic and inflammatory pattern than the adherent women. Overall, patients increased their intake of oligosaccharides; consistent with this, we observed with the pregnancy progression an enrichment in inferred metabolic pathways related with polysaccharide degradation, which in turn could be linked to the increased insulin resistance.

We found few associations between nutrient intake and microbial abundance. Fat intake was associated with *Alistipes* among Bacteroidetes, while *Roseburia* and *L*-*Ruminococcus* among the Firmicutes appeared related, though not significantly, with nutrients related to vegetable foods (oligosaccharides and fiber). This observation is in agreement with DNA-based studies evaluating the fecal microbiota during pregnancy in healthy overweight Finnish women at early pregnancy stage (17 week)^29^ as well as in normal-weight Norwegian women during the second trimester of pregnancy^30^. On the opposite, we observed a positive association between protein intake and *Faecalibacterium* which is in disagreement with previous studies^29,30^.

Research about gut microbiota composition and dietary intakes during pregnancy showed controversial results. Either no relationships between bacterial groups and dietary intakes^3,31^ or association between dietary fat and vitamin D with Proteobacteria increase^29^ and higher gut microbiota richness and lower abundance of Bacteroidaceae with increased dietary fiber intake have been reported^31^. Those findings confirm the great heterogeneity of results on this topic and highlight difficulties in the comparison of results from the studies, probably due to the different dietary habits, microbiota remodeling during pregnancy owing to hormonal changes and the additional insulin resistance determined by the presence of GDM.

Short-term changes in dietary pattern have been demonstrated to modulate quickly the microbiota composition. Rapid, but transient changes occur following dietary variations^8,10^, although longer and persistent modifications are needed to shape the human gut microbiota. In addition, the effects of diet on gut microbiota, rather than being direct, are hypothesized to be the consequence of the weight change and the subsequent variation in white adipose tissue inflammation and insulin resistance^26^.

In this study, we have assessed whether the modification of the dietary habits by dietary counselling during pregnancy can affect the gut microbiota composition. Even if arbitrary, the subdivision by dietary adherence distinguished women with greater increments of fasting glucose, insulin resistance and CRP values. During normal pregnancy a low grade of inflammation develops and GDM is a pro-inflammatory state^32^. Accordingly, we observed higher values of CRP at the pregnancy end. An imbalance of pro- and anti-inflammatory bacterial species have been proposed to trigger low-grade inflammation and insulin resistance in humans^18^. In particular *Faecalibacterium*, an anti-inflammatory commensal bacteria, significantly increased with pregnancy progression, but also increased in non-adherents, and is consistently reported to be more prevalent in individuals with higher bacterial richness^18^. It could be hypothesized that this increase could be a compensatory mechanism to counterbalance the pro-inflammatory state, potentially harmful for the fetus.

Indeed, we found a strong inverse relationship between *Faecalibacterium* abundance and fasting glucose values, supporting the well-known association between inflammation and dysmetabolism. Accordingly, *Faecalibacterium prausnitzii* resulted highly discriminant for the diagnosis of type 2 diabetes in metagenomic analyses^33,34^. Furthermore, these butyrate-producer bacteria have been found inversely linked to diabetes in human studies on fecal microbiota^35–38^.

In our patients, we observed a negative associations between diastolic blood pressure and *Rikenellaceae* and *Oscillospira*. *Rikenellaceae* is a butyrate producers, while *Oscillospira* is considered an enigmatic bacterial genus that has never been cultured, probably producing butyrate. Few available data support a beneficial role on human health^39^. Other studies found a protective role of *Odoribacter* (Bacteroidetes) on blood pressure in pregnant overweight women, and its capability to produce butyrate was mainly implicated in the maintenance of normal blood pressure^40^.

The strong direct associations that we found between the genus *Collinsella* and insulin/HOMA-IR values were in line with studies during pregnancy^7,41^ or not^37^ showing higher abundance of the lactate-producing *Collinsella* in type 2 diabetes mellitus. These bacteria can affect the metabolism by decreasing liver glycogenesis and playing pro-inflammatory effects^41^.

Insulin resistance was associated positively with *Erysipelotrichia* and negatively with *Blautia* in our patients. Very few human data are available on *Erysipelotrichia*, suggesting a relationship with inflammatory diseases for this class, which seems in a close relationship with the class of *Mollicutes*, which is in turn associated with many pathological human conditions like endotoxemia, obesity and insulin resistance^26,42^.

In our overweight GDM patients, the butyric acid-producing genus *Butyricimonas* was directly associated with insulin resistance. The possible role of this taxa in human diseases awaits further investigation, even if a positive association with mean arterial pressure has been detected^43^.

Changes in CRP values during the third trimester of pregnancy resulted directly associated with Sutterella. Even if we did not detect an overall increase in Proteobacteria during pregnancy, as other authors observed^3^, we found that *Sutterella*, a proteobacteria with known pro-inflammatory capacity, was associated with CRP increment across pregnancy. Consistent with this, the predicted metagenomes showed a correlation between *Sutterella* and KEGG genes associated with LPS biosynthesis. Gram-negative bacteria could produce inflammatory LPS triggering a pro-inflammatory state, a condition characterizing both type 2 diabetes and obesity^44^.

We also observed a correlation between LPS inferred KEGG genes and *Bacteroides*. In diabetic patients, LPS from a specie belonging to *Bacteroides* (*B. fragilis*) was reported to play a major pathogenic role^45^. *Bacteroides* is often associated with high fat-animal based diet^9^. Consistently, we found a *Bacteroides* reduction in adherents only, whose total and saturated fat intake, and CRP values decreased across pregnancy (Table 2). In addition, the metagenomic content of GDM patient was reported to be enriched of genes involved in LPS biosynthesis and in the regulation of blood glucose levels^13^.

At genus level, strong inverse relationships between *Blautia* and Hba1c and insulin resistance were observed. At sub-genus level, we observed a higher number of oligotypes belonging to the same species, even if only a few of them changed during the progression of the pregnancy, and controversial associations between *Blautia* and blood pressure and cholesterol values. A controversial role of *Blautia* in the human gut is reported. Several studies showed a direct association between *Blautia* and hyperglycemia^46,47^ but other studies reported that abundance of this taxon indicates a healthy gut, reduced inflammation and blood pressure values, diminished risk for type 1 diabetes and obesity, and increased survival^48,49^. Our results suggest a possible different strain-dependent effect on metabolism. The diversity at sub-genus-level is indeed well known to play a key role in establishing the interconnection between gut microbiome and host responses^50^. As recently observed by De Filippis and colleagues^51^, different oligotypes belonging to the same species showed different relative abundance and different correlation patterns with metabolomic data. Those authors suggested that different putative strains could have different impact on the host^51^.

The knowledge of the gut bacterial composition might allow the identification of subsets of women with different metabolic risks, owing to its role in the gestational pro-inflammatory status potentially contributing to the increased insulin resistance of pregnancy. This is a topic of great interest, also in consideration of the benefits of probiotic supplementation in the reduction of inflammation in women with GDM^52^, a condition well-known for exposing to an increased risk for chronic health conditions not only the mother but also her child.

One limitation of this study is the small sample size; nevertheless, the power of our study to detect differences in alpha diversity was 0.84 with α = 0.01. The fecal samples were used as proxies for the microbial content of the entire gastrointestinal tract; it is reasonable to consider that mouth and skin microbiota could vary too. The limitations of the food questionnaires must be recognized, even if these were widely used. Most of our patients had a very low fiber intake and consumed a high-fat diet; indeed, the dietary intakes of our patients resembled those of other pregnant women^31,53^, and this finding is in line with the well-known associations between GDM and unhealthy diet^1^. The lack of substantial difference in dietary intakes between enrolment and study end should be recognized as a limitation; we cannot exclude that a better adherence to the dietary recommendations could have resulted in greater differences between adherents and non-adherents. Nonetheless, it is noteworthy that already small dietary changes have been able to lead to statistical and clinical significant difference between groups, suggesting the importance of a healthy diet in these patients. Owing to the observational design of this study, the presence of unmeasured confounding factors cannot be excluded. Microbiota assessment through amplicon-based sequencing has several biases due to the PCR amplification step, while shotgun metagenomic sequencing identified significantly more bacterial species per read than the 16S method^54^. Correlations were performed by considering individual groups of bacteria independently from each other, therefore it was not possible to establish neither the causality nor the biological relevance of the reported relationships. Finally, the predictive metagenomic profiling was obtained from the bacterial abundance and was therefore a derived result.

In our overweight GDM patients, a shift in the microbiota composition with higher α-diversity, and numerous associations between the metabolic/inflammatory pattern and specific bacterial abundance were detected. If confirmed by further studies in larger sample, these results suggest that the development of strategies to modulate the gut microbiota might be the next step in order to impact on maternal and possibly fetal health and their future risk for metabolic diseases.

## Methods

### Patients recruitment

The participants were 50 patients with GDM consecutively recruited from the “Città della Salute e della Scienza” Hospital of Turin from April 2016. Each participant gave her written informed consent to participate in the study. The study protocol was approved by the Ethics Committee of the “Città della Salute e della Scienza” Hospital of Turin (approval 707/2016). All research was performed in accordance with relevant guidelines/regulations.

Inclusion criteria were: gestational age between 24–28 weeks, Caucasian race, GDM diagnosed by a 75 g oral glucose tolerance test (OGTT). Women who had the following criteria were excluded from the study: twin pregnancy, use of prebiotics/probiotics, antibiotics or any drug during pregnancy, any pathological conditions before or during pregnancy (known diabetes mellitus, hypertension, cardiovascular, pulmonary, autoimmune, joint, liver or kidney diseases, thyroid dysfunction, cancer, any other disease/condition), no compliance to the study protocol. All women were taking folic acid supplementation.

GDM was diagnosed by OGTT performed at 24–28 gestational weeks in the morning, after at least 8h-overnight fast, when the fasting plasma glucose was ≥92 mg/dL and/or 1 h post-OGTT glycemia ≥180 mg/dL and/or 2 h post-OGTT glycemia ≥153 mg/dL, according to international criteria^17^. In our cohort, all the patients with GDM routinely received dietary counselling and nutritional recommendations in line with guidelines (carbohydrates 45% total energy, rapidly absorbed sugars <10% total energy, proteins 18–20% total energy, fats 35% total energy, at least 20–25 g/day fibre intake, no alcohol)^17^. Furthermore, 30-min daily moderate exercise was recommended (i.e. brisk walking). Patients were instructed to self-monitor finger-prick capillary blood glucose at least 4 times per day. Insulin treatment was prescribed by the physicians in the presence of hyperglycemia, in accordance with guidelines^55^.

### Sample collection, anthropometric measurements and dietary information

Questionnaires, anthropometric values, fasting blood samples and stool samples were collected for all participants both at 24–28 weeks of gestational age at the time of GDM diagnosis (enrolment), and at 38 weeks, or before delivery, in the case of preterm delivery (study end). The researchers were in continuous contact with the patients, through weekly telephone contact. In this way, they were aware of the progress of pregnancy.

Stool samples were self-collected by the patients as previously described^27^. Briefly, the subjects were instructed on how to self-collect the samples, and all materials were provided in a convenient, refrigerated, specimen collection kit. Patients were provided with sterile containers to collect the feces (VWR, Milan, Italy). The fecal samples were collected at home and transferred to the sterile sampling containers using a polypropylene spoon (3 spoons of about 10 g) and immediately stored at 4 °C. The specimens were transported to the laboratory within 12 hours of collection at a refrigerated temperature. Containers were immediately stored at −80 °C for DNA extraction. No storage medium was used.

Participants completed a 3-day food record (2 weekdays and 1 weekend day) and the Minnesota-Leisure-Time-Physical Activity Questionnaire^56^ at enrolment and at the study end. Detailed information on how to record food and drink consumed by using common household measures was provided to all participants. Two dieticians checked all questionnaires for completeness, internal coherence and plausibility.

Data relative to pre-pregnancy weight was self-reported; weight, height, and arterial blood pressure (BP) were measured at time of enrolment, and weight and BP at the study end. Body weight was measured to the nearest 0.1 kg, and height was measured to the nearest 0.1 cm with a stadiometer (SECA model 711, Hamburg, Germany), with the participants wearing light clothes and no shoes. Arterial BP was measured from the left arm, in a sitting position, after at least 10 min of rest, with a mercury sphygmomanometer with appropriate cuff sizes (ERKA Perfect-Aneroid, Germany). Two measurements were taken by trained personnel with arm supported at heart level and the values reported were the means of the two. Glucose levels were self-measured by the patients by the BGSTAR® glucometer (Sanofi-Deutscland GmbH, Frankfurt, Germany). The average of the values measured 1-hour after each meal during the third trimester has been reported.

Babies were classed as large for gestational age (LGA) if their birthweights were >90^th^ percentile, considering neonatal anthropometric standards for Northern Italy^57^.

### Blood analyses

Serum glucose was measured by the glucose oxidase method (Sentinel Ch., Milan) with an intra-assay CV of 1.1% and an inter-assay CV of 2.3%. HbA1c levels were determined by a latex-based method (Sentinel, Milan, Italy). The intra-assay e inter-assay CVs were respectively 1.1–1.5% and 1.1–1.6%. Triglycerides and cholesterol were assayed by enzymatic colorimetric assays (Sentinel, Milan) with an intra-assay CV of 3.0% and an inter-assay CV of 3.5% for triglycerides and with an intra-assay CV of 2.2% and an inter-assay CV of 3.4% for cholesterol. HDL-cholesterol was determined by enzymatic colorimetric assay after precipitation of LDL and VLDL fractions using heparin-MnCl_2_ solution and centrifugation at 4 °C and it had an intra-assay variation CV of 2.5% and an inter-assay CV of 4.1%. Insulin was measured by a biotin labelled antibody-based sandwich enzyme immunoassay (LDN, Germany). The kit had a sensitivity of less than 1.8 U/mL and a range of 0–100 U/mL. The intra-assay and inter-assay CVs were respectively 1.8–2.6% and 2.9–6.0%. Serum CRP values were determined using a high-sensitivity-latex agglutination assay on HITACHI 911 Analyzer (Sentinel, Milan). The intra-assay and inter-assay CVs were 0.8–1.3% and 1.0–1.5%, respectively. All laboratory measurements were centralized.

BMI was calculated as weight divided for the square of height. The HOMA-IR was calculated according to the published algorithm^58^. Adherence to the given dietary recommendations was considered in the presence of all the following criteria: consuming at least 20 g/day fiber (or increasing fiber intake more than 50% than enrolment) and reducing sugars <10% of total energy and abolishing alcohol intake.

### Fecal DNA extraction

Nucleic acid was extracted from the feces collected. Total DNA from the samples was extracted using the RNeasy Power Microbiome KIT (Qiagen, Milan, Italy) following the manufacturer’s instructions. One microliter of RNase (Illumina Inc. San Diego. CA) was added to digest RNA in the DNA samples, with an incubation of 1 h at 37 °C. DNA was quantified using the QUBIT dsDNA Assay kit (Life Technologies, Milan, Italy) and standardized at 5 ng/μL.

### 16S rRNA amplicon target sequencing

DNA directly extracted from fecal samples was used to assess the microbiota by the amplification of the V3-V4 region of the 16S rRNA gene using the primers and protocols described by Klindworth *et al*.^59^. PCR amplicons were cleaned using Agencourt AMPure kit (Beckman Coulter, Milan, Italy) and the resulting products were tagged by using the Nextera XT Index Kit (Illumina Inc. San Diego. CA) according to the manufacturer’s instructions. After the 2nd purification step, amplicons products were quantified using a QUBIT dsDNA Assay kit (Life Technologies). Subsequently, equal amounts of amplicons from different samples were pooled. The pooled sample was run on an Experion workstation (Biorad, Milan, Italy) for quality analysis prior to sequencing. The sample pool (4 nM) was denatured with 0.2 N NaOH, diluted to 12 pM, and combined with 20% (vol/vol) denatured 12 pM PhiX, prepared according to Illumina guidelines. The sequencing was performed with a MiSeq Illumina instrument (Illumina) with V3 chemistry and generated 250 bp paired-end reads according to the manufacturer’s instructions.

### Bioinformatics analysis

Paired-end reads were first assembled using FLASH software^60^ with default parameters. Joint reads were further quality filtered (at Phred <Q20) using QIIME 1.9.0 software^61^ and short reads (<250 bp) were discarded through Prinseq^62^. Chimera filtering was performed through USEARCH software version 8.1^63^. Operational Taxonomic Units (OTUs) were picked at 97% of similarity threshold by UCLUST algorithms^64^ and centroids sequences of each cluster were matched to the Greengenes 16S rRNA gene database version 2013. After sequencing, a total of 2,100,009 raw reads (2 × 250 bp) were obtained. After joining, a total of 1,919,311 reads passed the filters applied with QIIME, with an average value of 23,406 ± 31,535 reads/sample and a sequence length of 457 bp. The rarefaction analysis and Good’s coverage, expressed as percentages, indicated that there was satisfactory coverage for all the samples (Good’s coverage average, 92%). In order to avoid biases due to the different sequencing depth, OTU tables were rarefied to the lowest number of sequences per sample (4078 reads/sample). The OTU table displays the higher taxonomy resolution that was reached; when the taxonomy assignment was not able to reach the genus, family name was displayed. Phylogenetic Investigation of Communities by Reconstruction of Unobserved States (PICRUSt) was used to predict abundances of KEGG orthologs (KO) based on 16S-based structure of the microbiota^65^. The KO abundance table was then collapsed at level 3 of the KEGG annotations in order to display the inferred metabolic pathways and the table was imported in *gage* Bioconductor package^66^ in order to carry out pathway enrichment analysis to identify biological pathways overrepresented or underrepresented between samples.

### Oligotyping analysis

In order to identify sub-OTUs populations, reads assigned to genera within *Ruminococcaceae* and *Lachnospiraceae* were extracted and entropy analysis and oligotyping were carried out^67^. Briefly, the extracted reads were then used to identify nucleotide positions that will explain the maximum amount of biological diversity across the samples utilizes Shannon entropy in order to identify positional variation to facilitate the identification of nucleotide positions of interest^67^. Only *Blautia* and *Roseburia* oligotypes showed a higher level of entropy and were the only two taxa able to be differentiated in sub-OTUs. After the first round of oligotyping, high entropy positions were chosen (-C option) 8, 9, 12, 223, 225, 247, 261, 282, 432, 433 for *Blautia*; while position 8, 9, 12, 245, 432, 433 and 434 were chosen for *Roseburia*. To reduce the noise, each oligotype was required to appear in at least 10 samples, occur in more than 1.0% of the reads for at least one sample, represent a minimum of 500 reads in all samples combined, and have a most abundant unique sequence with a minimum abundance of 100. BLASTn was used to query the representative oligotype sequences against the NCBI nr database, and the top hit was considered for taxonomic assignment.

### Statistical analysis

Gut microbiota α-diversity was assessed by Chao1 index, estimating the number of different taxa, and by Shannon diversity index, evaluating the taxa richness and evenness calculated using the diversity function of the vegan package^68^ in R environment (http://www.r-project.org). OTU table was used to build a principal-component analysis (PCA) as a function of the sampling time by using the made4 package of R. ADONIS and ANOSIM statistical test was used to detect significant differences in the overall microbial community by using the Weighted UniFrac distance matrices and the OTU table.

Not-normally distributed variables were presented as median (range interquartile). The individual differences between endof the study values minus baseline values were calculated (deltas). The delta median values were reported. Within-participant differences in bacterial richness and in the variables at enrolment compared with values at the pregnancy-end were evaluated by paired-sample t-test, or Wilcoxon matched pairs test, as appropriate. Differences between categorical variables were computed by chi-square test.

Differences in gut microbiota or oligotypes between adherents and non-adherents were calculated by t-Student test or Mann-Whitney test. Box plots represented the interquartile range between the first and the third quartile, with the error bars showing the lowest and the highest value. Pairwise Spearman’s non-parametric correlations were used to study the relationships between the relative abundance of microbial taxa abundance or oligotypes and dietary and metabolic variables, and between gut microbiota and inferred metabolic pathways. The correlation plots were visualized in R using the corrplot package of R.

Multiple regression analyses were performed to evaluate the associations between log-transformed microbial taxa abundance or oligotypes (dependent variable) and nutrient intakes after adjusting for age and weight (variables at baseline), or age and weight change (variables at the pregnancy end). The associations between blood pressure and laboratory variables (dependent variables) with OTUs were calculated by a multiple regression model, after adjusting for age and weight (variables at baseline) or age, weight change, and adherence to the given dietary recommendations (variables at the pregnancy end) (Statistica, ver. 7.0; StatSoft Inc., Tulsa, OK, USA).

Bonferroni’s correction for multiple comparisons was applied; a *P* value of 0.002 or lower was considered as statistically significant.

### Ethics approval and consent to participate

Each participant gave her written informed consent to participate in the study. The study protocol was approved by the Ethics Committee of the “Città della Salute e della Scienza” Hospital of Turin (approval 707/2016).

## Electronic supplementary material

Supplementary file

## Data Availability

All the sequencing data were deposited at the Sequence Read Archive of the National Center for Biotechnology Information (SRA accession number: SRP135886).
