# Supplementary material for: Changes in the gut microbiota composition during pregnancy in patients with gestational diabetes mellitus (GDM)
Source: Sci Rep. 2018 Aug 15;8:12216. doi: 10.1038/s41598-018-30735-9 (PMC6093919; doi:10.1038/s41598-018-30735-9)
Supplement: Supplementary file 1 — Supplementary file [file 41598_2018_30735_MOESM1_ESM.docx]

**Changes in the gut microbiota composition during pregnancy in patients with gestational diabetes mellitus (GDM)**

Ilario Ferrocino^1^, Valentina Ponzo^2^, Roberto Gambino^2^, Adriana Zarovska^2^, Filomena Leone^3^, Clara Monzeglio^4^, Ilaria Goitre^2^, Rosalba Rosato^5^, Angelo Romano^6^, Giorgio Grassi^2^, Fabio Broglio^2^, Maurizio Cassader^2^, Luca Cocolin^1^* and Simona Bo^2^*

^1^Department of Agricultural, Forest and Food Sciences, University of Turin, Italy

^2^Department of Medical Sciences, University of Turin, Italy

^3^Clinical Nutrition Unit, S. Anna Hospital, Città della Salute e della Scienza, Turin, Italy

^4^Gynecology and Obstetrics Unit, S. Anna Hospital, Città della Salute e della Scienza, Turin, Italy

^5^Department of Psychology, University of Turin, Turin, Italy

^6^SC Controllo Alimenti e Igiene delle Produzioni, Istituto Zooprofilattico Sperimentale PVL, Turin, Italy

**Fig. S1**. Average-linkage clustering based on the Spearman distance of fecal samples of GDM patients at enrolment (green bars) and at the study end (blue bars). Rows and columns are clustered by Ward linkage hierarchical clustering.


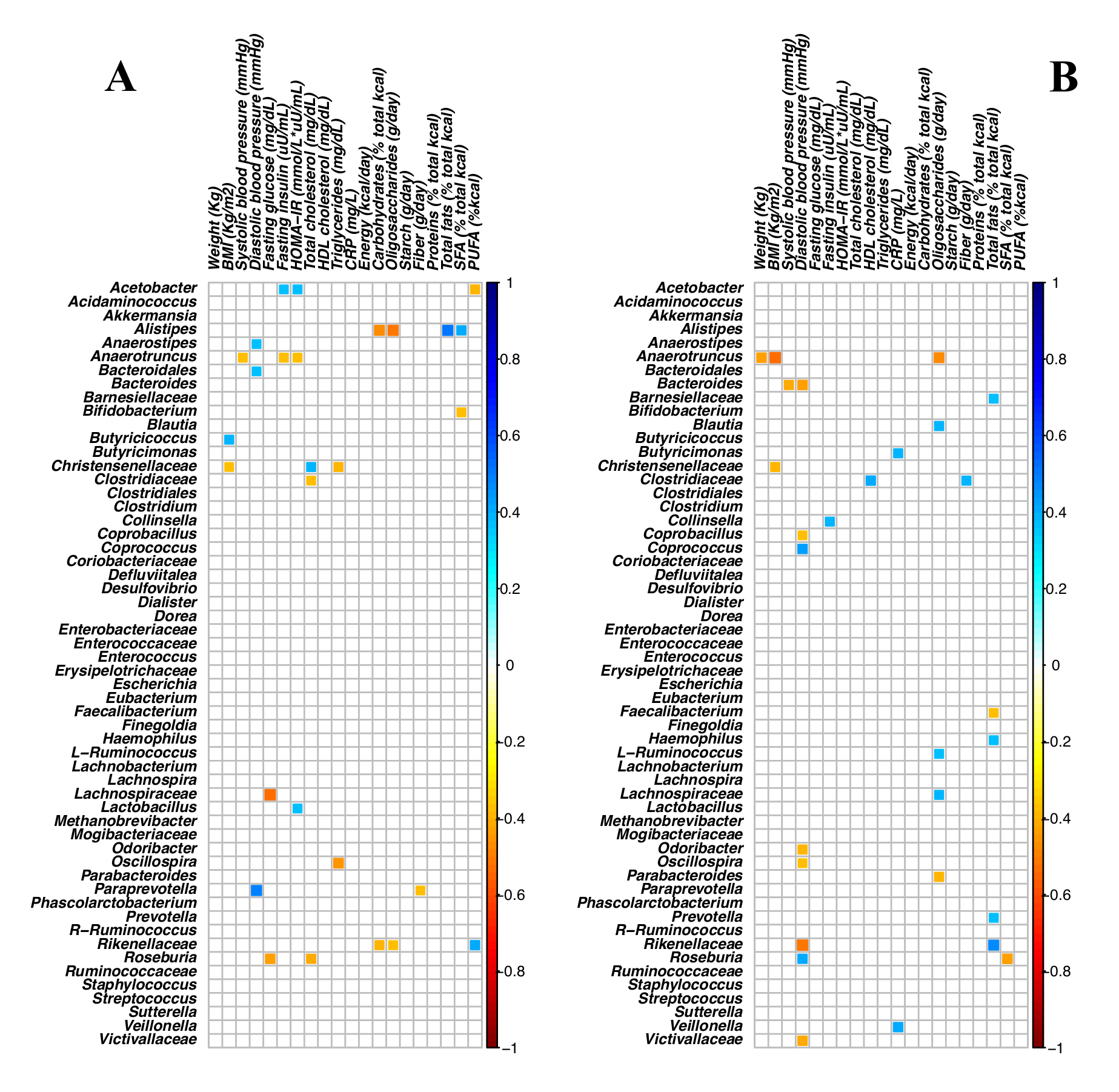


**Fig. S2.** Spearman’s rank correlation matrix of OTUs with > 0.2% abundance in at least 10 fecal samples, dietary information and blood variables. Strong correlations are indicated by large squares, whereas weak correlations are indicated by small squares. The colors of the scale bar denote the nature of the correlation, with 1 indicating a perfectly positive correlation (dark blue) and -1 indicating a perfectly negative correlation (dark red) between the two datasets. Only significant correlations (P <0.01) are shown. GDM patients at enrolment (plot A) or at study end (Plot B).


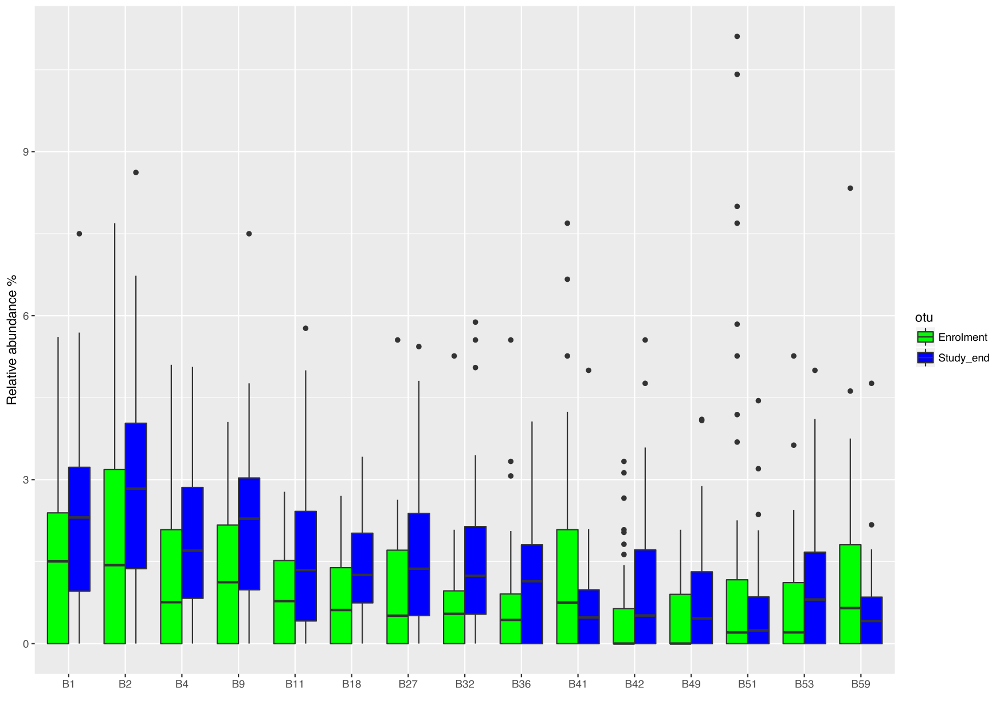


**Fig. S3.** Boxplots of differentially abundant Blautia oligotypes in fecal samples between GDM patients at enrolment (green bars) and at the study end (blue bars). Only oligotypes that significantly differed between enrolment and study-end fecal samples are displayed (P <0.01).


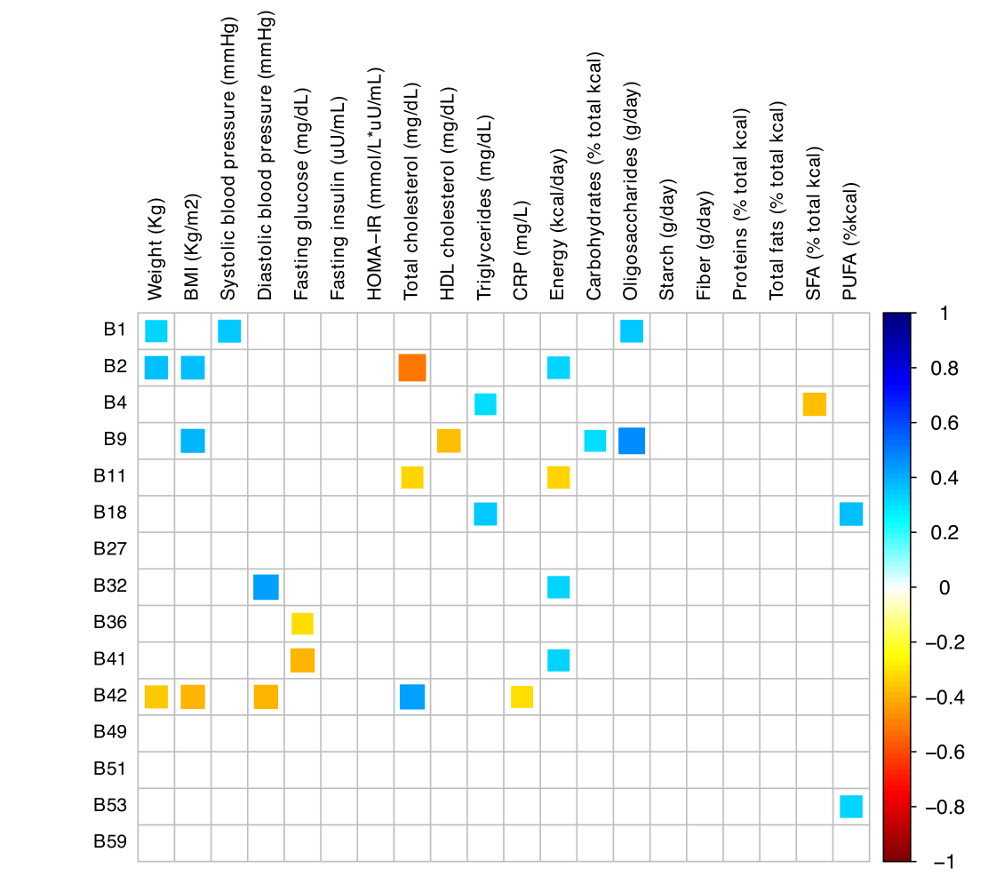


**Fig. S4**. Spearman’s rank correlation matrix of Blautia oligotypes abundance, nutrients, and blood variables. Strong correlations are indicated by large squares, whereas weak correlations are indicated by small squares. The colors of the scale bar denote the nature of the correlation, with 1 indicating a perfectly positive correlation (dark blue) and -1 indicating a perfectly negative correlation (dark red) between the two datasets. Only significant correlations (P <0.01) are shown.

**Fig. S5**. Heat plot showing Spearman’s correlations between OTUs occurring at 0.2% in at least 10 samples and predicted metabolic pathways, filtered for KO gene sample presence >10 in at least 10 samples, related to amino acid, lipid, cofactors and vitamins, energy and carbohydrate metabolism. Rows and columns are clustered by Ward linkage hierarchical clustering. The intensity of the colors represents the degree of correlations between the OTUs and KO as measured by the Spearman’s correlations.
